# The Marlin Wonder

**Published:** 1897-11

**Authors:** 


THE MARLIN WONDER.—The hot artesian wells of Marlin, Falls County, Texas, are a wonder. The main well is over 3000 feet deep, the deepest artesian well in the world; said to be. The temperature of the water is 140° F. The Sanitarium Company, of Marlin, have erected a magnificent hotel, the "Arlington," and a sanitarium at the wells, and thousands of invalids are going there for the benefit of these hot wells. Every facility is afforded for the use of the water, and a skillful physician superintends the administration of baths for the treatment of disease. Many persons whom we have met recently have used these baths, and express themselves as well pleased with the results.

